# Isolation and characterisation of *Methylocystis* spp. for poly-3-hydroxybutyrate production using waste methane feedstocks

**DOI:** 10.1186/s13568-020-01159-4

**Published:** 2021-01-06

**Authors:** Bashir L. Rumah, Christopher E. Stead, Benedict H. Claxton Stevens, Nigel P. Minton, Alexander Grosse-Honebrink, Ying Zhang

**Affiliations:** BBSRC/EPSRC Synthetic Biology Research Centre (SBRC), School of Life Sciences, University of Nottingham, University Park, Nottingham, NG7 2RD UK

**Keywords:** Methanotrophy, *Methylocystis* species, Poly-3-hydroxybutyrate, Bioplastic, Biogas

## Abstract

Waste plastic and methane emissions are two anthropogenic by-products exacerbating environmental pollution. Methane-oxidizing bacteria (methanotrophs) hold the key to solving these problems simultaneously by utilising otherwise wasted methane gas as carbon source and accumulating the carbon as poly-3-hydroxybutyrate, a biodegradable plastic polymer. Here we present the isolation and characterisation of two novel *Methylocystis* strains with the ability to produce up to 55.7 ± 1.9% poly-3-hydroxybutyrate of cell dry weight when grown on methane from different waste sources such as landfill and anaerobic digester gas. *Methylocystis rosea* BRCS1 isolated from a recreational lake and *Methylocystis parvus* BRCS2 isolated from a bog were whole genome sequenced using PacBio and Illumina genome sequencing technologies. In addition to potassium nitrate, these strains were also shown to grow on ammonium chloride, glutamine and ornithine as nitrogen source. Growth of *Methylocystis parvus* BRCS2 on Nitrate Mineral Salt (NMS) media with 0.1% methanol vapor as carbon source was demonstrated. The genetic tractability by conjugation was also determined with conjugation efficiencies up to 2.8 × 10^–2^ and 1.8 × 10^–2^ for *Methylocystis rosea* BRCS1 and *Methylocystis parvus* BRCS2 respectively using a plasmid with ColE1 origin of replication. Finally, we show that *Methylocystis* species can produce considerable amounts of poly-3-hydroxybutyrate on waste methane sources without impaired growth, a proof of concept which opens doors to their use in integrated bio-facilities like landfills and anaerobic digesters.

## Keypoints

- *Methylocystis rosea* BRCS1 was isolated from a lake while *Methylocystis parvus* BRCS2 was isolated from a bog, both in England.
- Both species showed normal growth and PHB accumulation on landfill and anaerobic digester gas which contain trace contaminants speculated to be  inhibitory to growth.
- *Methylocystis parvus* BRCS2 showed the highest PHB accumulation 55.7 ± 1.9% PHB of cell dry weight when grown using landfill gas as methane source.

## Introduction

Methane (CH_4_) is the second most abundant greenhouse gas (GHG) produced by human activity with a global warming potential up to 105 times higher than CO_2_ over a 20-year period (Rodhe 1990; Shindell et al. 2009). Methane is emitted from a variety of anthropogenic and non-anthropogenic sources including wetlands, natural gas exploration sites and landfill sites (Boeckx et al. 1996; Allen et al. 2013; Zhang et al. 2017). High quality biogas from Anaerobic Digester (AD) and landfill sites is currently economically used for energy production (Allen et al. 2013). However, biogas with low methane content is often flared with the aforementioned environmental impact (EPA 2011). To improve incentive for biogas capture, new technologies for utilisation of the gas need to be explored.

As the only known biological sink for atmospheric methane, methane-oxidizing bacteria are largely responsible for balancing methane flux in the environment through oxidation of methane for a source of carbon and energy (Anthony 1982). The use of methanotrophs to produce platform chemicals, single cell protein or biopolymers has high economic potential (Strong et al. 2016). Biopolymer production in particular has received renewed societal and industrial interest with reports of petrochemical, non-biodegradable plastics polluting the environment and earth’s oceans (Derraik 2002; Eriksen et al. 2014). Typically, plastic compounds cannot be degraded by microorganisms. Rather they disintegrate into ever smaller fragments called microplastics. Microplastics have been found throughout the marine ecosystem and pose possible adverse effects on ecological and human health (Cole et al. 2013; Wright and Kelly 2017). Poly-3-hydroxybutyrate (PHB) is a short-chained poly-hydroxyalkanoate (PHA) with mechanical properties comparable to isotactic polypropylene (PP) and polyethylene (PE), with the advantage that it is biodegradable (Tokiwa et al. 2009; Yeo et al. 2018). PHB is produced by type II methanotrophs during nutrient limitation and it serves as a source of reducing equivalents (Asenjo and Suk 1986; Wendlandt et al. 2001; Listewnik et al. 2007; Pieja et al. 2011). Therefore, the utilisation of PHB producing methanotrophic organisms grown on comparably cheap or waste sources of methane such as AD or landfills could represent a consolidated solution to two major environmental problems from anthropogenic activity.

Factors such as ability to utilise methane feedstock and PHB accumulation capability of the chosen methanotrophic chassis need to be taken into consideration when selecting a bacterial strain. Most studies reported to date on methanotrophic PHB production have mainly focused on the use of pure methane, natural gas or artificial biogas as substrate, leaving the renewable sources of CH_4_ (biogases) open to investigation (Pieja et al. 2011; Listewnik et al. 2007; López et al. 2018). Biogas from anaerobic digesters and landfills consist primarily of a mixture of methane, carbon dioxide (CO_2_) and nitrogen, with traces of toxic compounds such as hydrogen sulphide (H_2_S), siloxanes and aromatic and halogenated compounds (Rasi et al. 2007). Also, biogas composition is highly dependent on waste composition, temperature and moisture among other factors, and can thus vary between different AD facilities and landfill sites (Rasi et al. 2007). Here we investigate the effect of biogas from three landfill sites and four different AD sources on growth and PHB production of two newly isolated strains of *Methylocystis* species, and compare their performance against the type strain *Methylocystis parvus* OBBP.

## Materials and methods

### Bacterial strains and culture conditions

All strains used in this study are listed in Additional file 1: Table S1. Methanotrophic strains were cultured in liquid Nitrate Mineral Salt (NMS), in dNMS medium (5 times diluted NMS medium with H_2_O) or on solid plates of NMS or dNMS supplemented with 1.5% Agar Bacteriological (Thermo Scientific, UK) (Whittenbury et al. 1970). Unless otherwise stated, liquid cultures were grown at 30 °C in serum bottles capped with rubber stoppers with a 5:1 headspace to culture ratio and headspace was adjusted to a 2:1 molar oxygen to methane ratio with 0.5 bar overpressure. Cultures on solid medium were grown at 30 °C in anaerobic Oxoid jars (Thermo Scientific, UK) by addition of methane to the headspace. Methanotrophs were stored at − 80 °C on microbeads (Microbank™ Bacterial and Fungal Preservation System, Pro-Lab Diagnostics, UK) according to the supplier’s instructions and revived on solid medium before inoculation into liquid medium.

### Isolation of methanotrophs

Environmental samples leading to isolation of *Methylocystis rosea* BRCS1 were collected from a recreational lake at the University of Nottingham campus (52° 56′ 13.9′′ N 1° 11′ 29.4′′ W) on 16th of March 2015. Enrichment started within 24 h of sampling. The sample was vortexed and centrifuged at 1000 rpm for 1 min. 10 µL of the supernatant was added to 60 mL serum bottles containing 10 mL dNMS media. Serum bottles were incubated at 30 °C and 150 rpm for 25 days with a ratio of air:CH_4_:CO_2_ of 76:20:4. All samples were processed in duplicates. Samples exhibiting visible growth were sub-cultured by adding 10 µL of the enrichment culture to fresh 60 mL serum bottles containing 10 mL of dNMS media and incubated as above. After five days of growth, the samples were serially diluted up to 10^–5^ and 100 µL of each dilution was spread on dNMS agar plates. Plates were incubated in Oxoid jars as described above.

On day 5 and 21, growth on plates was analysed. Colonies growing on agar were resuspended in 15  µL Nuclease Free Water (NFW) and re-spread on dNMS agar plates. Once colonies formed, they were analysed for Methane Monooxygenase (MMO) gene presence by PCR using specific primers (*pmoA* and *mmoX*) and PCR products were Sanger sequenced (Eurofins Scientific, UK) (Bourne et al. 2001). Colonies testing positive for MMO genes (*pmoA* and/or *mmoX*) were purified through multiple rounds of growth in liquid culture starting from serial dilutions followed by growing to single colony on dNMS agar. After several rounds of such purification, vitamins in the medium were omitted to inhibit growth of non-methanotrophic bacteria. A pure culture of BRCS1 was obtained after further rounds of purification.

Samples leading to isolation of *Methylocystis parvus* BRCS2 were obtained from a bog in Moseley UK (52° 26′ 10.5" N 1° 51′ 55.0" E) and stored at room temperature overnight. 3 g of solid bog samples (gravel sediment and bog sediment) were homogenised in 27 mL dNMS media supplemented with 10 μM CuSO_4_∙7H_2_O using a vortex. Sediments in the samples were settled and supernatant was serially diluted with supplemented dNMS medium to 10^–7^. 11 mL of each dilution was transferred to a 60 mL serum bottle. Serum bottle headspace was adjusted to 20:80 CH_4_:air ratio and samples were incubated at 30 °C for five weeks shaking at 150 rpm.

Samples were visually analysed for growth after five weeks and highest dilutions per sample showing growth were plated on dNMS agar plates. Single colonies were further purified after two rounds of liquid culturing and plating, as explained above. At this stage, colonies were analysed by PCR and Sanger sequencing as described above and MMO positive colonies were further purified by extinction-dilution as follows: colonies were resuspended in dNMS media omitting vitamins, diluted to 10^–7^ in 96-well plates and incubated at 30 °C and 200 rpm in the gas-tight box CR1601 (EnzyScreen, NL) for two weeks. From the highest dilutions showing visible growth, 5 µL were streaked on dNMS agar plates and grown for 10 days. This process was repeated until pure isolates were obtained.

Purity of isolates were tested by observation of cells under Phase Contrast Microscope (PCM) and by PCR of 16S rRNA with primers U515f: GTGYCAGCMGCCGCGGTA and U1071r: GARCTGRCGRCRRCCATGCA (Wang and Qian 2009). Growing liquid cultures of isolates were spread on LB agar, 10% LB agar, Tripticase Soy Agar (TSA) (Sigma-Aldrich, UK) and 10% TSA. The plates were incubated at 30 °C under normal atmospheric pressure. Absence of growth on rich media suggested pure methanotroph cultures.

### Conjugation of methanotrophs

Conjugation was carried out based on modifications of the method used by Martin and Murrell (1995). 5 mL of *E. coli* S17-1 λ pir harbouring plasmids pMTL90882 or pMTL71401 (Dr Muhammad Ehsaan, University of Nottingham, unpublished) was grown overnight in LB media containing 50 µg/mL kanamycin. Absorbance (OD_600_) of the grown culture was measured and used to calculate the volume required to get 1 mL of *E. coli* donor at OD_600_ of 1. The calculated volume of *E. coli* was pipetted in 2 mL Eppendorf tubes and washed three times with NMS media to remove the antibiotics by spinning at 8000 rpm for three minutes. After the third wash, the *E. coli* donor pellet was mixed 1:1 with recipient methanotrophic isolates. The mixture was spun down at 8000 rpm for three minutes and resuspended in 50 μL of NMS which was spotted on NMS agar supplemented with 0.5% yeast extract. The conjugation was incubated at 30 °C with methane for 48 h. The conjugation spot was scraped with a plastic loop and resuspended in 1 mL NMS which was serially diluted to 10^–7^. Each dilution was spotted in triplicates on NMS agar with 50 µg/mL kanamycin for plasmid retention and 25 µg/mL nalidixic acid as selection against *E. coli*. Dilutions were also spotted on LB media containing 50 µg/mL kanamycin to calculate number of donor cells. NMS media Plates were incubated with methane at 30 °C for two weeks after which transconjugants were enumerated to calculate conjugation efficiency (transconjugants/donor cell) (Phornphisutthimas et al. 2007). LB media plates were incubated at 37 °C overnight to calculate number of donor cells.

### Growth of methanotrophs on anaerobic digester and landfill gas

Biogas samples from anaerobic digesters 1–4 (AD1–AD4) and landfill gas samples 1- 3 (LG1–LG3) were collected in 2 L Teddlar bags (Sigma-Aldrich, UK) in different locations around the UK on different dates (Table 1). The AD gases were collected from Staffordshire at different dates while the LG were collected from different sites around the East Midlands of England. Bulk gas composition was measured using Trace GC (see below) while trace gas composition for AD1 and AD2 was measured by Lucideon Ltd in Staffordshire to understand potential trace contaminants.

**Table 1:** Gas composition measured by GC for CH_4_, CO_2_, O_2_ and by Lucideon Ltd for trace contaminants H_2_S, H_2_ and NH_3_

Sample	Source location	Date of sampling	CH_4_ (%)	CO_2_ (%)	O_2_ (%)	N_2_ (%)	H_2_S (ppm)	H_2_ (ppm)	NH_3_ (mg/m^3^)
AD1	Staffordshire	17/12/17	49.0	40.3	2.1	7.7	94.0	0	2.9
AD2	Staffordshire	16/01/18	58.6	33.4	2.1	7.8	462.0	0	> 0.7
AD3	Staffordshire	26/02/19	62.4	40.5	0.5	0.0	682.0	23.0	0
AD4	Staffordshire	26/02/19	58.0	38.0	0.4	0.0	34.0	16.0	0
LG1	East Midlands	07/01/2019	52.2	37.3	0.4	10.1	n/a	n/a	n/a
LG2	East Midlands	08/01/2019	45.6	31.5	0.4	22.5	n/a	n/a	n/a
LG3	East Midlands	08/01/2019	51.9	38.7	0.4	9.0	n/a	n/a	n/a

*n/a* not applicable (not measured)

The first set of experiments involved growth on AD1 and AD2 to determine if methanotrophs could grow on AD biogas. Methanotrophs were grown in duplicates on AD1 and AD2 gases as follows: 9 mL of methanotroph culture at an OD_600_ of 0.02 was added to 165 mL serum bottle together with 75% air and 25% of either AD1, AD2 or CH_4_ as carbon source. Samples were incubated at 30 °C for 4 days.

### PHB analysis

PHB accumulation in methanotrophs was achieved using a method similar to preliminary assays described in Additional file 1. In short, one 250 mL serum bottle with 35 mL NMS medium was inoculated from colonies growing on plates and grown for several days with pure methane. This culture was used to inoculate main cultures (35 ml NMS in 250 ml serum bottles) to OD_600_ 0.05 and gassed with the respective biogas and air. Biogas was added to result in 0.65 mM methane and air to make up at least a 2:1 molar ratio and to result in 1.5 bar pressure in the serum bottle. This culture was grown for three days, re-gassing every day, before the grown cells were resuspended in NMS media without nitrogen source (potassium nitrate) to trigger PHB accumulation. The culture was then re-gassed daily for another 3 days and then harvested for PHB analysis. Daily re-gassing during PHB accumulation phase was not incorporated in experiments involving anaerobic digester gases.

After three days of PHB accumulation, the cell cultures were pelleted and freeze-dried overnight using the Thermo Micromodulyo Freeze Dryer (Thermo Scientific, UK). The pellets were transferred to a pre-weighed 2 mL Eppendorf and weighed again. These pellets were then transferred to 16 mm diameter round-bottom screw cap centrifuge tube. In a fume cupboard, 100 μL of 1 mg/mL benzoic acid solution in 1-propanol was added. This was followed by 4 mL of 25% concentrated hydrochloric acid in 1-propanol. The glass tubes were then heated at 100 °C for two hours. After cooling, 4 mL of deionised water was added, and each tube was vortexed for 30 s to cause phase separation. The top layer was discarded, 4 mL of deionised water was added, and each tube was vortexed again for 30 s. The top layer was discarded for the second time and 1 mL of the bottom layer was transferred into a GC snap vial cap which was analysed on an Agilent 6890 N Series gas chromatograph, equipped with an Agilent 7983 autosampler, an Agilent 5973 MS detector and a J & W DB-wax column (20 m × 0.18 mm, 0.18 mm film thickness). Injection temperature of 250 °C was applied with standard single split insert with a glass wool packing. The oven program following 1 µL injection was as follows: 5 min hold at 60 °C, 20 °C/min to 240 °C, and hold for 6 min. Split injections were made with 10:1 split ratio. Hydrogen carrier gas with constant flow control was used at 0.6 mL/min. MS analysis was in scanning mode from 40 to 500 m/z produced with EI auto ionization, the MS solvent delay was 3 min and no additional voltage was applied to the electron multiplier.

## Results

### Isolation of methanotrophs

Samples for methanotroph isolation were taken from two UK locations, a bog in Moseley and a recreational lake at the campus of The University of Nottingham. Isolation and purification procedures for samples of each location varied as outlined in “Materials and methods”, and both resulted in the isolation of pure methanotrophic cultures. Purity of both isolates was confirmed by absence of non-methanotrophic bacterial growth on rich media without methane addition. Phase Contrast Microscopy (PCM) also confirmed single bacterial morphology. The 16S rRNA of the isolate from the lake sample showed 100% similarity to *Methylocystis rosea* GW6 and was subsequently designated as *Methylocystis rosea* BRCS1. The isolate from the bog sample shared 100% 16S rRNA sequence similarity to *Methylocystis parvus* OBBP and was designated *Methylocystis parvus* BRCS2.

### Morphology of isolated methanotrophs

**Fig. 1:**
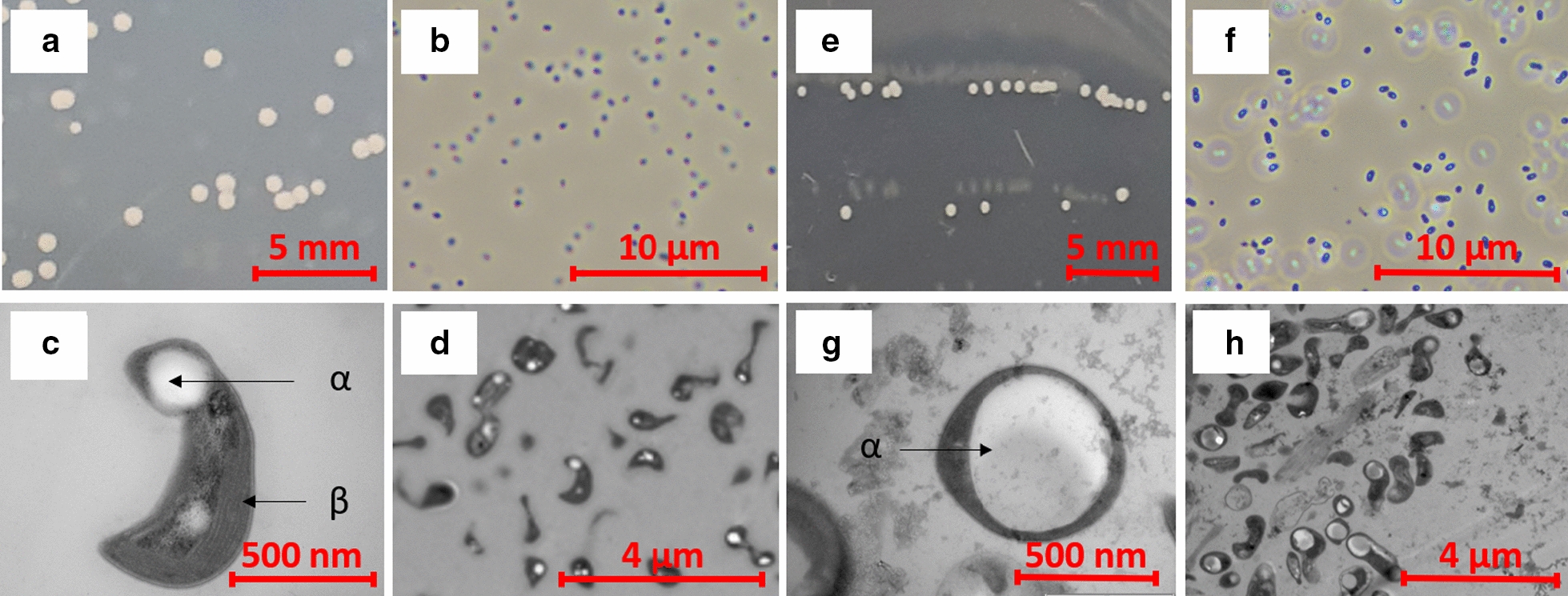
Morphological studies of BRCS1 on the left (**a**–**d**) and BRCS2 on the right (**e**–**h**). **a**, **e** Respective colony morphology on NMS agar plates. **b**, **f** Phase-contrast photomicrograph at ×100 magnification. **c**, **g** Transmission electron photomicrograph of single cells at ×60,000 magnification. **d**, **h** Transmission electron photomicrograph at ×4200 and ×8200 magnification, respectively. **c** The dumbbell-like shape with a head- and tail-like part. α) The head part includes a light-transmissive granule thought to consist of the storage compound PHB. β) The tail part show striata which are believed to be the ICM. **g** A single coccus-shaped cell with a big light-transmissive granule (α) also thought to consist of the storage compound PHB

Morphology of the isolated methanotrophs was studied based on colony formation on agar plates, Phase Contrast Microscopy (PCM) and Transmission Electron Microscopy [TEM (method in Additional file 1)]. *Methylocystis rosea* BRCS1 colonies appeared cream-coloured after 10 days of growth on NMS agar plates. After 2–4 weeks, the cream colour gradually converted to and remained pink (Fig. 1a). Colonies were concave and grew up to 3 mm in diameter. Single cells appeared oval according to PCM imaging (Fig. 1b). However, TEM revealed polymorphic cells with a prominent head-like structure and a thin to thick tail-like structure (Fig. 1c, d). The tail-like structure resembles a rod-like appendage in some cells and a stalk in others. It is speculated that the cells use the tail for adherence on surfaces or to one another as it is the case in other stalked species (Curtis 2017). Measuring cell length and width from TEM images using ImageJ revealed the whole length of a cell including stalk to be 116 ± 19 nm and the width of the head-like structure to be 53 ± 10 nm (mean ± SD, n = 20) (Pérez and Pascau 2013). The head-like structure comprised of a prominent white granule which is suspected to be PHB, used for redox balancing (Fig. 1c). Striations visibly circumscribing the cell periphery are thought to be the Intracytoplasmic Membrane (ICM) (Fig. 1c).

*Methylocystis parvus* BRCS2 colonies were concave and had a cream appearance (Fig. 1e). Prolonged incubation (more than a month) was observed to lead to drying out and solidifying of the colonies. Single cells had a short, thick, dumbbell-like morphology with a length of 139 ± 20 nm and a width of 65 ± 12 nm (measured from TEM images using ImageJ, mean ± SD, n = 14). BRCS2 presented a prominent white storage granule suspected to be PHB and can be observed in most of the cells (Fig. 1g).

### Conjugation efficiency of *Methylocystis rosea* BRCS1 and Methylocystis parvus BRCS2

**Fig. 2:**
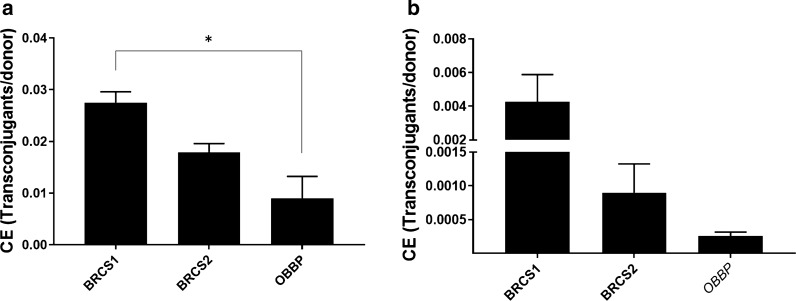
Conjugation efficiencies of BRCS1 and BRCS2 compared to established strain *M. parvus* OBBP (OBBP) with two differing plasmids. **a** Plasmid pMTL90882 harbouring the ColE1 replicon and **b** pMTL70401 harbouring the pBBR1 replicon. Statistically significant difference is indicated with an asterisk compared to the OBBP control and calculated by t-test as indicated in the text. All samples n = 2 with error-bars representing SEM. Graphs made using GraphPad Prism

Efficiency of DNA transfer is an important characteristic of a newly isolated strain with biotechnological potential. Therefore, conjugation efficiency of the newly isolated strains compared to the established laboratory strain *M. parvus* OBBP was determined using two plasmids with different origins of replication (ORI), ColE1 and pBBR1 (Lovett et al. 1974; Antoine and Locht 1992). Efficiency of conjugation was measured as number of transconjugants per donor cell (TC/DC). Plasmid pMTL90882 (ColE1) conjugation efficiency was significantly higher for strain BRCS1 (2.8 × 10^–2^ ± 1.5 × 10^–3^) compared to OBBP (8.9 × 10^–3^ ± 3.3 × 10^–3^) tested by Unpaired t-test (p = 0.035) (Fig. 2a). Conjugation Efficiency of pMTL71401 featuring the pBBR1 ORI was 4.3 × 10^–3^ ± 1.2 × 10^–3^, 9 × 10^–4^ ± 3 × 10^–4^ and 2.6 × 10^–4^ ± 3.8 × 10^–5^ for BRCS1, BRCS2 and OBBP respectively (mean ± SEM, n = 2). pMTL71401 (pBBR1) conjugation efficiency does not differ significantly from the new isolates compared to OBBP tested by Unpaired t-test (Fig. 2b).

### Genomic DNA sequencing and analyses

Chromosomal DNA of *M. rosea* BRCS1 and *M. parvus* BRCS2 was extracted and sequenced as described in Additional file 1. Both species were found to carry two autonomous replicating plasmids. Annotation was carried out by NCBI with accession number of CP044328, CP044329 and CP044330 for BRCS1 chromosome and plasmids, corresponding to 3,386,331 bp; 195,485 bp and 213,640 bp respectively. GC content was calculated as 62.67%. BRCS2 chromosome and plasmids accession numbers are CP044331, CP044332 and CP044333, corresponding to sizes of 4,075,934 bp; 248,223 bp and 204,886 bp respectively. GC content was calculated as 63.35%.

The analysis file received from PacBio was sent to Rebase to reveal the Restriction Modification (RM) systems of BRCS2 which was shown to have both type II and III systems (Roberts et al. 2015). BRCS2 was shown to possess RM systems on its genome and mega plasmid that recognise the sequence GANTC, GATC and CTCGAG (Additional file 1: Figure S1 and Table S2). The information obtained from Rebase can be used for increasing genetic transformation efficiencies of isolates. One area where high transformation efficiencies can be desirable is when making transposon mutant libraries in which hundreds of thousands of transconjugants are required which is easier to achieve with high transformation efficiencies.

The completed genome sequences were analysed for genes responsible for carrying out cellular tasks such as DNA repair, homologous recombination and PHB metabolism. DNA repair genes play important roles during genome editing, an area planned for investigation in future studies. Genes involved in Non-Homologous End Joining (NHEJ) during DNA repair were found in isolated strains (Additional file 1: Table S3). *ykoV* and *ykoU* were present in BRCS1 while in BRCS2, the genes involved in NHEJ during DNA repair were *ligD* and the Ku protein genes. In *M. parvus* (OBBP), *ligD* and Ku protein genes were also present. PHB metabolism genes found in *M. rosea* BRCS1 were also found in *M. parvus* BRCS2 (similar to *M. parvus* OBBP) including *phbA*, *phbB* and *phbC.* In addition, an esterase family of PHB depolymerase was found in BRCS1 (Additional file 1: Table S3). Understanding genes involved in PHB metabolism can enable genetic engineering of strains with better PHB accumulation properties.

### Phylogenetic tree and genomic alignment of novel isolates

**Fig. 3:**
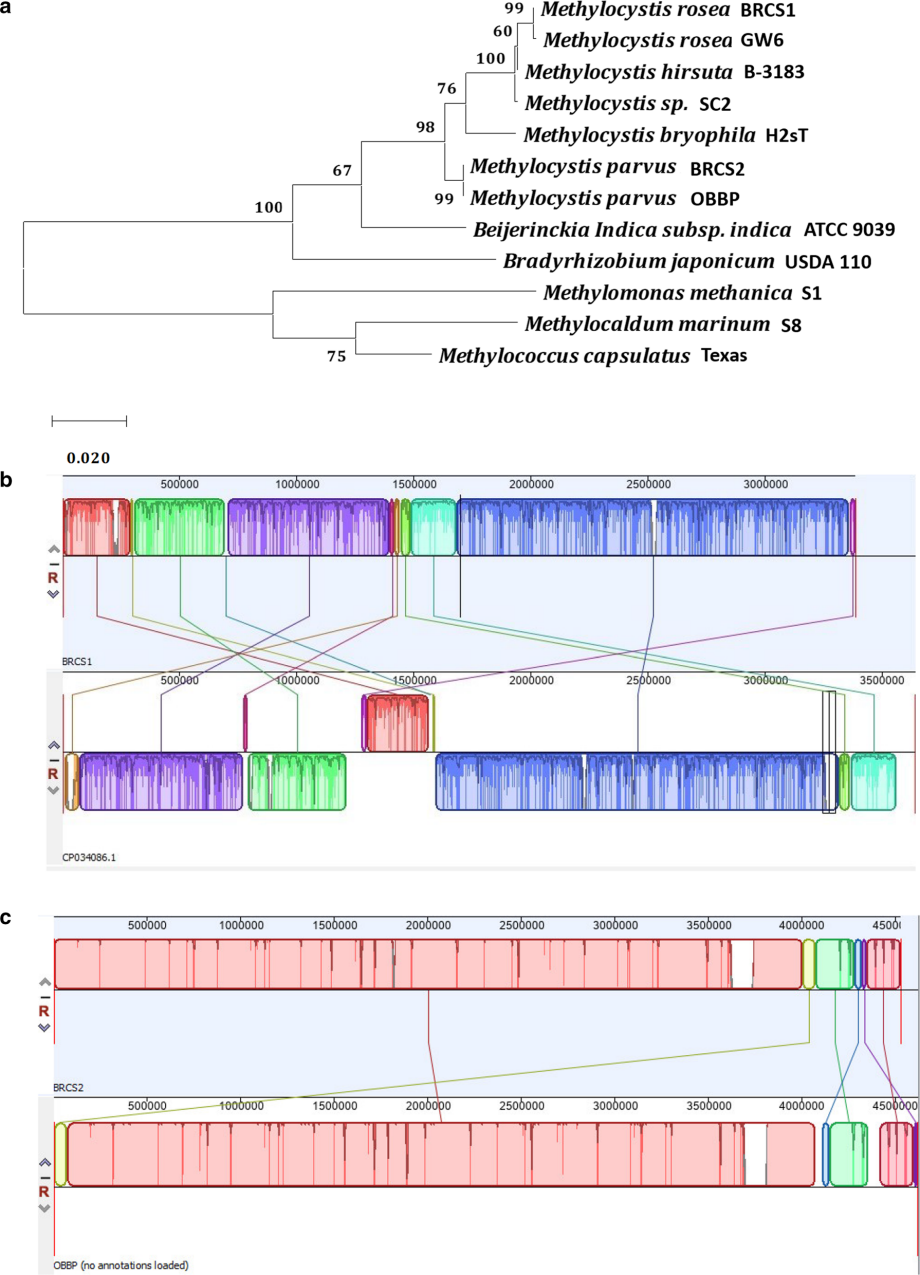
Phylogenetic tree comparing isolated strains of *Methylocystis* species with related species and whole genome alignment of isolated strains with closest relatives based on 16S rRNA similarity. **a** The tree is drawn to scale, with branch lengths measured in the number of substitutions per site. This analysis involved 12 nucleotide sequences. Codon positions included were 1st + 2nd + 3rd + Noncoding. Bootstrap method was used as test of phylogeny with 1000 number of Bootstrap Replications. There were a total of 1547 positions in the final dataset. Evolutionary analyses were conducted in MEGA X9. b/c) The alignment shows structural and single nucleotide similarity between isolated strains and the closely related strains they were compared to. With Average Nucleotide Identity of 94.96%, *M. rosea* BRCS1 had significant levels of structural and single nucleotide differences when compared to *M. rosea* GW6 with accession number CP034086.1. **a** There was significantly less structural and single nucleotide difference between *M.* parvus BRCS2 and *M. parvus* OBBP with Average Nucleotide Identity of 99.99% (**b**)

Phylogenetic analysis was carried out to determine the relationship of isolated strains to known strains of methanotrophs and distantly related species (Fig. 3a). *M. rosea* BRCS1 was closely related to the already published *M. rosea* GW6, both of which fell under the same clade. The same relationship was observed between *M. parvus* BRCS2 and the type strain *M. parvus* OBBP. The close relationship was expected due to 100% similarity of their 16S rRNA sequence.

Genomic DNA alignment provided more insight into the similarity of isolates to closely related strains in terms of genomic structure and nucleotide similarity. The comparison of *M. rosea* BRCS1 to *M. rosea* GW6 genome revealed relatively high level of structural dissimilarity (Fig. 3b). Additionally, there was high level of nucleotide dissimilarity when genes of both strains were compared with Average Nucleotide Identity of 94.96%. This was unexpected considering both species had 100% 16S rRNA sequences, suggesting the 16S rRNA does not necessarily imply whole genome resemblance. On the other hand, *M. parvus* BRCS2 and *M. parvus* OBBP showed high similarity in terms of genomic structure and nucleotide comparison which was expected as a result of the 100% 16S rRNA similarity (Fig. 3c). The calculated Average Nucleotide Identity was 99.99%.

### Growth characteristics of *Methylocystis rosea* BRCS1 and *Methylocystis parvus* BRCS2

Two crucial media components are important for growth of methanotrophs–nitrogen and carbon source. As such, we set out to test various sources of both growth components.

### Growth on different nitrogen sources

Growth was tested on various potential nitrogen sources (potassium nitrate, ammonium chloride, asparagine, glutamine, ornithine, aspartate, lysine, and putrescine) by measuring final OD_600_ after 14 days of growth. Both strains BSRC1 and BSRC2 grew best with potassium nitrate as nitrogen source. While BRCS1 can grow on ammonium chloride, glutamine and ornithine, final OD_600_ is reduced to about 50% whereas final OD_600_ of BRCS2 is reduced to roughly 20% on these nitrogen sources compared to growth on potassium nitrate (Additional file 1: Figure S2).

### Growth on methanol

**Fig. 4:**
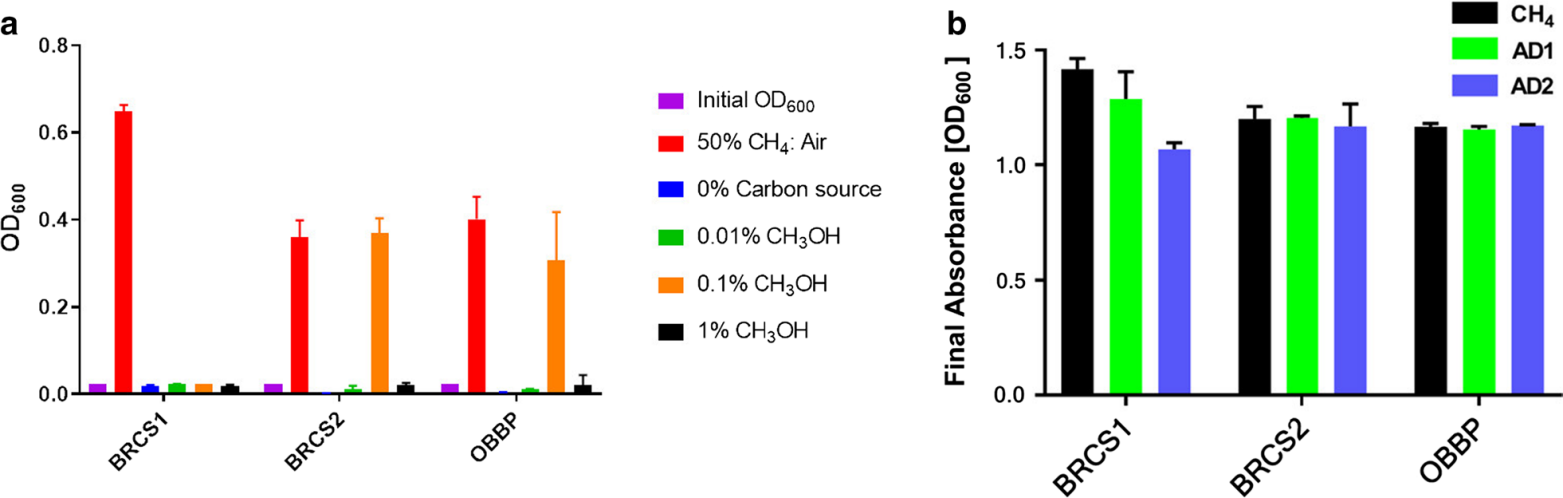
Growth of methanotrophs on different carbon sources. **a** Growth of methanotrophs with different CH_3_OH concentrations. Growth in CH_4_ was used as control. There was significant difference across all absorbance values measured with p < 0.0001 using one-way ANOVA. **b** Growth characteristics of new isolates BRCS1 and BRCS2 were compared with control strain OBBP on biogases from anaerobic digesters (AD1/2). Final absorbance at OD_600_ was compared after 4 days of growth (n = 2). Graphs made using GraphPad Prism

After determining the suitability of potassium nitrate as nitrogen source in the media, both strains were tested on the ability to grow on methanol instead of methane as sole carbon source. After 10 days of shaking and incubation at 30 °C in 65 mL serum bottles, *M. parvus* BRCS2 like its closest evolutionary relative *M. parvus* OBBP was able to grow with 0.1% methanol vapour, while BRCS1 was not able to grow with the methanol concentrations tested in the range 0.01–1% (Fig. 4a).

### Methanotrophic growth on biogas from anaerobic digesters (AD)

#### Anaerobic digester gas composition

Bulk composition of biogases from anaerobic digesters (AD1–AD4) and gases from landfill sites (LG1–LG3) were measured by trace GC (Table 1). Methane content ranged from 49% in AD1 to 62.4% in AD3. Carbon dioxide content was measured at 32% in LG2 and up to 40.5% in AD3. All samples contained traces of oxygen which are assumed to originate from gas exchange through the Teddlar gas collection bags and not from the original sample as those environments are expected to be anaerobic. The rest of the gas composition is made up of nitrogen, hydrogen sulphide and other trace gases. Ammonia, hydrogen sulphide and siloxane composition of gases AD1 and AD2 were assessed and found to be minimal (Table 1 and Additional file 1: Table S4).

The ability of the isolates to grow on renewable forms of biogas from anthropogenic sources such as anaerobic digesters (AD) and landfills is crucial if they are to be utilised for industrial biotechnology. Therefore, an initial experiment was conducted to investigate growth of the novel strains on un-purified gas from AD which carries potentially toxic contaminants such as ammonia, siloxanes, hydrogen sulphide and aromatics as well as halogenated compounds (Rasi et al. 2007). Growth of BRCS1, BRCS2 and OBBP using two biogas samples (AD1 and AD2) was compared to growth on CH_4_ as control (Fig. 4b). No significant growth difference was observed of strains growing on pure CH_4_ compared to growth on AD gas sources, tested by two-way ANOVA with Dunnett’s post hoc test (p > 0.05). These findings suggest that for these species, contamination of up to 1.1 mg/m^3^ siloxane (Additional file 1: Table S4) and 682 ppm H_2_S as well as 2.9 mg/m^3^ ammonia are non-problematic in methanotrophic culturing as speculated, opening the possibility of integrated methanotrophic facilities at AD sites.

#### PHB production in methanotrophs

**Fig. 5:**
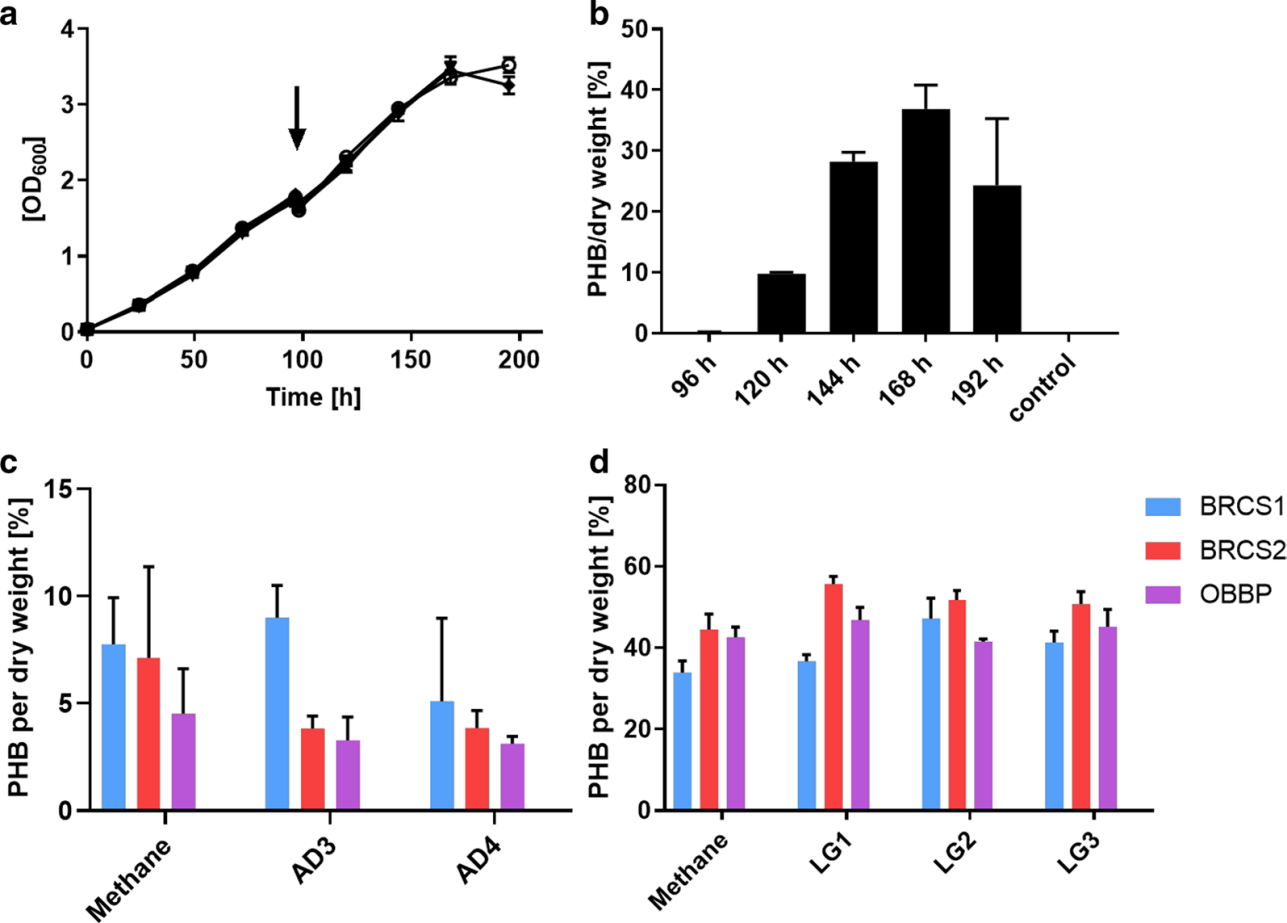
Poly-3-hydroxybutyrate (PHB) production of new methanotroph isolates. **a** Preliminary experiment to test optimal PHB accumulation with type strain OBBP. Growth was measured during the pre-culture which grew supplemented with nitrate (NO^-^_3_) which was harvested after 96 h (indicated by and arrow) and resuspended in medium without nitrate. Samples with black symbols and control (grown with nitrate for 192 h) with empty symbol. **b** PHB accumulation over time during nitrate limited phase (n = 2). PHB production of new methanotroph isolates compared to PHB producing type strain *M. parvus* OBBP and PHB negative strain *Methylococcus capsulatus* Bath from two individual experiments (**c** and **d**). **c** Test of methanotrophic PHB production on biogas from AD unfiltered (AD3) and filtered (AD4). **d** Test of methanotrophic PHB production on biogas from three different landfills. OBBP served as PHB producing positive control of the type II methanotrophs and Bath as negative control. The bar chart shows PHB per cell dry weight accumulated in a 35 mL culture after 3 days growth followed by three days growth without nitrate. All samples n = 3 with error-bars representing SEM. Graphs made using GraphPad Prism

Having shown that contaminants in biogas from anaerobic digesters are non-toxic to the new isolates and the type strain *M. parvus* OBBP, it was further tested if the isolates produce the biopolymer poly-3-hydroxybutyrate (PHB) when biogases from landfill sites (LG1/2/3) and anaerobic digesters (AD3/4) are used as CH_4_ source. It has been shown that PHB accumulation can be triggered by nitrogen limitation in the medium (Wendlandt et al. 2001; Listewnik et al. 2007; Pieja et al. 2011). Hence, we adopted a two-stage growth and production method to maximise PHB accumulation. Peak PHB accumulation was observed on day 3 of the second growth stage (nitrate-limiting conditions). Incubation lasting longer than 3 days under nitrate-limiting conditions led to decreased PHB yield as experiments showed (Fig. 5a, b). For this initial experiment, the type strain *Methylocystis parvus* OBBP was used with pure CH_4_ serving as the source of carbon.

Once it was established that PHB accumulation under nitrate-limiting conditions peaked on day 3 in the preliminary experiment, subsequent experiments harvested cell cultures for PHB assays on day 3 of incubation on nitrate-free media. Four strains (*M. rosea* BRCS1, *M. parvus* BRCS2, *M. parvus* OBBP and *M. capsulatus * Bath which served as control) were then grown using the respective landfill and AD gases as CH_4_ source. The results showed that all type II methanotrophs tested were able to grow and accumulate PHB using pure CH_4_ as well as landfill and AD gas as source of CH_4._ It was observed that PHB accumulation of all strains when grown on landfill gas was higher than when grown on AD gas. Although there is a possibility that landfill gas components can trigger higher PHB accumulation, it is more likely as a result of the method used during PHB accumulation of the separate experiments. The results further show that *M. parvus* BRCS2 produced the most PHB per dry cell weight (55.7 ± 1.9%) compared to the other strains on all gases tested. When grown on AD gases, *M. parvus* BRCS2 also performed better in producing PHB per dry cell weight (24.8 ± 2.0%) than other strains. The control strains performed as expected. *M. capsulatus* Bath as a type I methanotroph acting as negative control did not produce any PHB, while *M. parvus* OBBP acting as positive control produced up to 46.8 ± 3.2% dry cell weight of PHB when grown on landfill gas (Fig. 5c, d). This is close to the highest value of 50.3 ± 3.3% obtained in the study carried out by Pieja et al. (2011). When PHB accumulation was compared between filtered (AD3) and unfiltered (AD4) AD gas, no significant difference (P = 0.253) was observed for all strains using one-way Anova. The unfiltered AD gas has a H_2_S content about 20 times higher than the filtered gas.

## Discussion

Isolation of methanotrophs was carried out in this study which resulted in two pure *Methylocystis* species. The characteristics of these isolates including morphology, physiology, genetics, genomics and the ability to accumulate PHB on biogas were investigated.

Isolation of methanotrophs from bog and lake samples was achieved without immediate addition of methane when the environmental samples were collected. Further processing of the samples took place after 24 h suggesting ability of methanotrophs to survive short term in the absence of methane. In fact, separate experiments leading to the isolation of a *Methylomonas* species and a novel strain of *Methylococcus capsulatus* were carried out after storage of the initial samples for four months at 4 °C (unpublished data). Previous studies reported survival of *Methylosinus trichosporium* OB3b after 10 weeks of CH_4_ starvation (Roslev and King 1994). These observations suggest that it is not essential to collect samples planned for methanotroph isolation under methane-enriched conditions. This potentially eases the process of sample collection for future isolations.

Isolation of methanotrophs was done according to protocols established previously with some adjustments aiding the purification of the strains presented here (Hoefman et al. 2012). Firstly, samples were cyclically alternated between growth in liquid culture and on agar plates. This alternation on the one hand allowed quick purification of desired methanotrophs from non-methanotrophic bacteria which can feed on methanotrophic metabolites such as acetate, formate and lactate (Whittenbury et al. 1970). On the other hand, some non-methanotrophs can grow on polysaccharides which make up agar, therefore switching from agar to liquid media potentially eliminated these contaminants (Payton and Roberts 1976; Imran et al. 2016). Furthermore, extinction dilution culturing was employed when transferring to liquid medium which further benefitted purification (Hoefman et al. 2012). The other important adjustment made to previous studies was the omission of vitamins in the defined NMS medium, potentially reducing the number of contaminating organisms auxotrophic for these vitamins. A disadvantage of this approach is the simultaneous loss of auxotrophic methanotrophs. However, isolation of methanotrophic species not requiring expensive vitamins in the medium (such as the isolates presented here) can be advantageous for subsequent industrial applications.

The newly isolated strains showed similar colony morphology at the outset of culturing on agar plates which manifests in round, cream coloured colonies. However, after about two weeks of growth, *M. rosea* BRCS1 transitioned to a pink colour potentially due to biosynthesis of carotenoids as was observed in other methanotrophic isolates (Leadbetter and Foster 1958). This hypothesis is further substantiated by the identification of the operon *crtBCDL* responsible for carotenoid biosynthesis in the genome of BRCS1. A subset of these genes, not including *crtD* were identified in BRCS2 as well, however the pathway does not seem active or is incomplete without *crtD*, suggested by the lack of pigment synthesis.

All strains could be transformed with plasmids carrying ColE1E and pBBR1 replicons via bacterial conjugation, with ColE1 replicon having a higher conjugation efficiency. The superiority of pMTL90882 over pMTL71401 was not surprising as ColE1 is a high copy number replicon (approximately 40 copies per chromosome in *E.coli*) compared to pBBR1 which has a low copy number (approximately 5 copies per chromosome in *E.coli*) (Jahn et al. 2016). The ability to accept heterologous plasmid DNA is advantageous for novel strains with biotechnological applicability. This enables designed genetic manipulation that can increase flux along desired biosynthetic pathways for products such as PHB. Furthermore, the complete genome sequence of the isolated strains offers more insight and explanation of some of the characteristics observed and suspected. Already, key genes have been mentioned involved in PHB metabolism and DNA repair pathways. The presence of mega plasmids was revealed in *M. parvus* BRCS2 which is likely present in *M. parvus* OBBP sequenced in 2012 but was not observed probably due to the sequencing technology at the time (del Cerro et al. 2012). This finding is crucial as it points towards greater understanding of the industrially relevant and widely studied *M. parvus* species. Mega plasmids can be used for plasmid addiction systems which enable industrial biotechnology applications of genetically engineered microorganisms.

With full 16S rRNA sequence from the genome of all strains, a more accurate phylogenetic analysis was possible. The phylogenetic tree constructed placed all type II methanotrophs such as *Methylocystis* species closer to one another compared to type I *methanotrophs.* This is not unreasonable, especially when taking into consideration that type II methanotrophs such as *Methylocystis* species which are *alphaproteobacteria* have a different process of carbon assimilation compared to type I methanotrophs like *Methylococcus capsulatus* which are methanotrophs *gammaproteobacteria* (Hanson and Hanson 1996). Further insight into the distant evolutionary ancestry between *alphaproteobacteria* and *gammaproteobacteria* is provided by evidence hinting that members of the family *Methylocystaceae* which are *alphaproteobacteria* have not always had the ability to oxidise methane. This ability is likely the result of lateral gene transfer from a methanotrophic *gammaproteobacteria* (Tamas et al. 2014).

Two sources of macronutrients are essential for normal growth of methanotrophs, nitrogen and carbon source. As such the effect they have on growth was investigated. The importance of investigating various nitrogen sources cannot be overemphasized because nitrogen starvation is directly linked to PHB accumulation. Furthermore, the choice of nitrogen source during methanotrophic growth phase was shown to influence PHB accumulation (Rostkowski et al. 2013). Potassium nitrate used throughout this study and in the commonly used NMS media was shown to be the best at supporting growth of the strains tested (Whittenbury et al. 1970).

Both strains of *M. parvus* were able to grow on methanol. The growth of *M. parvus* OBBP in methanol was already demonstrated in a previous study (Hou et al. 1979). However, it was important to verify this characteristic in the isolated strains *M. rosea* BRCS1 and *M. parvus* BRCS2. The ability of *M. parvus* BRCS2 to grow on methanol is noteworthy since this allows handling of the organism in places where methane atmosphere is not an option such as liquid handling robots. It can furthermore seamlessly slot into a methanol-based economy as proposed by Olah et al. (2006).

Investigating biogas sources is crucial because the ability of methanotrophs to grow can be influenced by the chemical composition of the biogas sources. For example, acetylene commonly found in natural gas was shown to inactivate the soluble methane monoxygenase used for methane oxidation in *Methylococcus capsulatus* (Prior and Dalton 1985). The successful growth of *Methylocystis* species on two different sources of AD gas (AD1 and AD2) provided the foundation and supporting evidence for further investigation of methanotrophic growth and PHB accumulation using other AD and landfill biogas sources. Additionally, the findings suggest that trace contaminants are non-problematic in methanotrophic culturing. Biogas from AD and landfill sites are currently mostly used for electricity generation but are flared or vented especially when CH_4_ concentration is low (Cashdollar et al. 2000; Tollefson 2016). In such circumstance, methanotrophs offer the possibility to utilise methane from low quality biogas.

In type II methanotrophs such as *M. parvus* OBBP, PHB is suspected to play a role in redox balancing and its accumulation usually manifests when nitrogen becomes limiting (Pieja et al. 2011). In this study, PHB accumulation was stimulated by nitrate starvation where nitrate-free media was used to incubate already grown cultures of methanotrophs. PHB accumulation peaked on day 3 of incubation in nitrate-free media as shown by (Asenjo and Suk 1986). PHB accumulation was higher when landfill gas was used as source of CH_4_. Although there is a possibility that landfill gas components can trigger higher PHB accumulation, it is more likely as a result of the method used during PHB accumulation of the separate experiments. Whereas daily air replenishment was carried out during PHB accumulation phase when methanotrophic strains were grown on landfill gas, that was not the case when methanotrophs were grown on AD gas. This suggests a positive influence of oxygen on PHB accumulation which was replenished daily when landfill gas was used as a source of CH_4_. The negative effect of oxygen limitation on PHB accumulation has been previously reported (Zhang et al. 2019).

The unfiltered AD gas has H_2_S content about 20 times higher than the filtered gas which was the only significant difference observed with the limited analysis conducted. The absence of significant H_2_S effect on PHB accumulation was also reported by López et al. (2018). However, the study conducted by López et al. (2018) significantly differs from ours considering synthetic biogas was used. Additionally, the study used *Methylocystis hirsuta,* a different species in the *Methylocystis* genus.

The highest PHB content per cell dry weight was measured to be 55.7 ± 1.9% with *M. parvus* BRCS2 on LG1. Similar yields were found in optimised bioreactor cultures which suggests a good performance of the isolate (Listewnik et al. 2007). However, as high as 60% PHB accumulation in *M. parvus* OBBP was reported by Rostkowski et al. (2013) although ammonium chloride was used as nitrogen source during the growth phase.

PHB can be harvested and used to make bioplastic which is not only from biological source but also biodegradable, a differentiation not always made in sustainability research (Mekonnen et al. 2013). The CO_2_ produced by the methanotrophs can be captured easily as fermenter off-gas and recycled in greenhouses for the growth of plants or in secondary fermentation by algae or syngas fermenting bacteria, further aiding mitigation of greenhouse gases (Mortensen 1987; Sayre 2010; Bengelsdorf 2013).

## Supplementary Information

**Additional file 1. **Supplementary information providing additional methods and results. Methods and results include: Bacterial strains and plasmid used in this study (Table S1); Method and result for next generation sequencing analysis; Rebase analysis of restriction patterns (Figure S1 and Table S2); Comparison of genes shared by isolates (Table S3); Method of Phase Contrast and Transmission Electron Microscopy; Method of phylogenetic tree analysis and whole genome alignment; Growth on different nitrogen sources (Figure S2); Siloxane composition of AD gases (Table S4); Method of preliminary PHB accumulation assay.

## Data Availability

The data that support findings in this study are openly available in NCBI as referenced in Additional file 1 of this study. All strains are deposited in NCIMB (National Collection of Industrial, Food and Marine Bacteria https://www.ncimb.com/) with the following accession numbers: NCIMB 15262 *Methylocystis parvus* BRCS2; NCIMB 15263 *Methylocystis rosea* BRCS1.
