# Supplementary material for: Isolation and characterisation of Methylocystis spp. for poly-3-hydroxybutyrate production using waste methane feedstocks
Source: AMB Express. 2021 Jan 6;11:6. doi: 10.1186/s13568-020-01159-4 (PMC7788130; doi:10.1186/s13568-020-01159-4)
Supplement: Supplementary file 1 — Additional file 1. Supplementary information providing additional methods and results. Methods and results include: Bacterial strains and plasmid used in this study (Table S1); Method and result for next generation sequencing analysis; Rebase analysis of restriction patterns (Figure S1 and Table S2); Comparison of genes shared by isolates (Table S3); Method of Phase Contrast and Transmission Electron Microscopy; Method of phylogenetic tree analysis and whole genome alignment; Growth on different nitrogen sources (Figure S2); Siloxane composition of AD gases (Table S4); Method of preliminary PHB accumulation assay. [file 13568_2020_1159_MOESM1_ESM.docx]

Isolation and characterisation of *Methylocystis* spp*.* for poly-3-hydroxybutyrate production using waste methane feedstocks

Bashir L. Rumah‡, Christopher E. Stead‡, Benedict H. Claxton Stevens, Nigel P. Minton, Alexander Grosse-Honebrink, and Ying Zhang*

BBSRC/EPSRC Synthetic Biology Research Centre (SBRC), School of Life Sciences, University of Nottingham, University Park, Nottingham NG7 2RD, UK.

‡ These authors contributed equally to the work

*Corresponding author: Ying Zhang: ying.zhang[@nottingham.ac.uk](mailto:mrznpm@exmail.nottingham.ac.uk)

**Additional Information**

**10 Pages**

Methods and results of:

Bacterial strains and plasmid used in this study (Table S1)

Method and result for next generation sequencing analysis

Rebase analysis of restriction patterns (Figure S1 and Table S2)

Comparison of genes shared by isolates (Table S3)

Method of Phase Contrast and Transmission Electron Microscopy

Method of phylogenetic tree analysis and whole genome alignment

Growth on different nitrogen sources (Figure S2)

Siloxane composition of AD gases (Table S4)

Method of preliminary PHB accumulation assay

**Bacterial strains and plasmids**

**Table S1**. Bacterial strains and plasmid used in this study.

| Name | Abbreviation | Properties | Source |
| --- | --- | --- | --- |
| *Escherichia coli* Top10 | Top10 | Cloning of plasmids used in this study. | Thermo Fisher Scientific, UK |
| *E. coli* S17-1 λ pir | S17-1 | Conjugation of plasmids into methanotrophic recipients | ^1^ |
| *Methylococcus capsulatus* (Bath) ATCC 33009 | Bath | Type I methanotroph wild type | ATCC |
| *Methylocystis parvus* (OBBP) NCIMB L 11129 | OBBP | Type II methanotroph wild type | NCIMB |
| *Methylocystis rosea* BRCS1 SBRC5822 | BRCS1 | Methanotroph isolate from recreational lake Nottingham, UK | This study |
| *Methylocystis parvus* BRCS2 SBRC5824 | BRCS2 | Methanotroph isolate from bog in Mosley, UK | This study |
| pMTL71401 |  | Modular plasmid for methanotrophs carrying a pBBR1 replicon, a multiple cloning site and kanamycin resistant cassette | SBRC Nottingham |
| pMTL90882 |  | Modular plasmid for methanotrophs carrying a ColE1 replicon, *trfA* encoding the replication initiation protein, *oriT* origin of transfer, *oriV* origin of vegetative replication, a multiple cloning site and a kanamycin resistant cassette. | SBRC Nottingham |

**Next Generation Sequencing (NGS) analysis**

Genomic DNA was extracted using phenol:chloroform:isoamyl alcohol gDNA extraction method (Sambrook et al., 1989). DNA quantity and purity was analysed by gel electrophoresis and Qubit Fluorometric Quantification (ThermoFisher Scientific, UK) according to supplier’s instructions. Genomic DNA libraries for Illumina sequencing were prepared using Nextera XT Library Prep Kit (Illumina, San Diego, USA) following the manufacturer’s instructions with slight modifications as follows: two nanograms of DNA were used as input, and PCR elongation time was increased to 1 min from 30 seconds. Illumina sequencing was carried out on an Illumina HiSeq using a 250 bp paired end protocol (MicrobesNG, UK). Reads were trimmed from adapters using Trimmomatic 0.30 with a sliding window quality cut-off of Q15 (Bolger et al., 2014). De Novo assembly was performed using SPAdes version 3.7, and contigs were annotated using Prokka 1.11 (Bankevich et al., 2014; Seemann, 2014). PacBio sequencing of isolated samples was carried out on a PacBio RS II system (Genome Quebec, CA). Library of sheared large inserts was created and sequenced using Pacbio SMRTcell. Contig assembly was done using the PacBio Hierarchical Genome Assembly Process HGAP workflow (Chin et al., 2013). Assembled PacBio genome was circularised and imported to CLC genomics Workbench version 10. Trimmed Illumina reads were imported and paired in CLC genomics, after which they were mapped to PacBio assembly for Single Nucleotide Polymorphism (SNPs) correction. SNP-corrected consensus sequence was submitted to NCBI for annotation.

Absence of RNA was confirmed by agarose gel electrophoresis and gDNA quantity was evaluated by fluorometric quantitation (Qubit). For BRCS1, the PacBio sequencing generated a total of 102977 raw subreads of average length 9904 bp using 1 SMRT Cell(s) in a PacBio RS II sequencer. A total of 106696 raw subreads of average length 10770 bp using 1 SMRT Cell(s) in a PacBio RSII sequencer was obtained for BRCS2.

PacBio contigs assembly for BRCS1 resulted in HGAP coverage cut-off (X) of 30 and HGAP length (b) cut-off of 17888 with estimated reads coverage of 253. There was a total of 4 contigs with N50 value of 3408803 bp and GC content of 62.67. Illumina reads were 446510 with mean coverage of 47.26. For BRCS2, contig assemble resulted in HGAP coverage cut-off (X) of 30 and HGAP length (b) cut-off of 17737 with estimated reads coverage of 245. There was a total of 3 contigs with N50 value of 4097387 bp and GC content of 63.35. Illumina reads were 468386 with mean coverage of 46.64.

**Rebase analysis of restriction patterns**

De Novo assembled genomes from Illumina and PacBio data were send to Rebase for restriction system analysis (Roberts and Vincze, 2009).


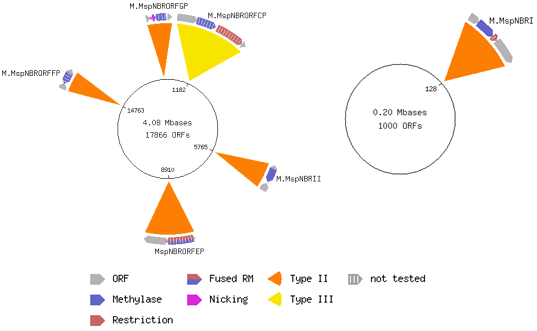


**Figure S1**. Restriction modification system of BRCS2 on genome (left) and second mega plasmid (right). Only one type II RM system is present on the mega plasmid while type II and III is present in the genome

**Table S2**. Putative *M. parvus* BRCS2 RM systems and predicted recognition sequence.

| Type | Gene | Predicted Recognition Sequence | Location | Nucleotide # |
| --- | --- | --- | --- | --- |
| II | M.MspNBRII | GANTC | genome | 1315067-1316356 |
| II | MspNBRORFEP |  | genome | 2022083-2024881 |
| II | M.MspNBRORFFP | GATC | genome | 3367098-3368321 c |
| II | V.MspNBRORFGP |  | genome | 4025438-4025851 c |
| II | M.MspNBRORFGP |  | genome | 4025856-4027121 c |
| III | M.MspNBRORFCP |  | genome | 270030-272033 |
| III | MspNBRORFCP |  | genome | 272044-275073 |
| II | M.MspNBRI | CTCGAG | plasmid | 25768-27519 |
| II | MspNBRIP | CTCGAG | plasmid | 27509-28273 |

**Table S3**. Comparison of genes shared by isolates sequenced in this study

| **Methanotrophs** | *M. rosea* (BRCS1) | *M. parvus* (BRCS2) | *M. parvus* (OBBP) |
| --- | --- | --- | --- |
| **DNA ligase genes for NHEJ** |  |  |  |
| *ligA* | ✓ | ✓ | ✓ |
| *ligD* | X | ✓ | ✓ |
| *ligD* Rv0938/MT0965 | ✓ | ✓ | ✓ |
| *ykoV* | ✓ | ✓ | ✓ |
| **DNA repair proteins for NHEJ** |  |  |  |
| *Ku* | X | ✓ | ✓ |
| *ykoU* | ✓ | X | X |
| **Homologous recombination genes** |  |  |  |
| *recBCD* | X | X | X |
| recombinase A | ✓ | ✓ | ✓ |
| *recF* (DNA replication and repair protein) | ✓ | ✓ | ✓ |
| *recG* ATP dependent DNA helicase | ✓ | ✓ | ✓ |
| *recJ* (Single stranded DNA-specific exonuclease) | ✓ | ✓ | ✓ |
| *recN* (DNA repair protein | ✓ | ✓ | ✓ |
| *recO* (DNA repair protein) | ✓ | ✓ | ✓ |
| *recQ* (ATP dependant DNA helicase) | ✓ | ✓ | ✓ |
| Recombination protein *RecR* | ✓ | ✓ | ✓ |
| *radA* | ✓ | ✓ | ✓ |
| **Polyhydroxybutyrate metabolism genes** |  |  |  |
| *phBC1* - Poly-beta hydroxybutyrate polymerase | ✓ | ✓ | ✓ |
| PHB/PHA accumulation regulator DNA-binding domain | ✓ | ✓ | ✓ |
| *phbA* Acetyl-coa Acetyltransferase | ✓ | ✓ | ✓ |
| *phbB* Acetoacetyl-Coa reductase | ✓ | ✓ | ✓ |
| *phbC2* - Poly-beta hydroxybutyrate polymerase | ✓ | ✓ | ✓ |
| *phb* depolymerase C terminus | ✓ | ✓ | ✓ |
| Esterase PHB depolymerase | ✓ | X | X |
| *phaE* Poly(R) - hydroxyalkanoic synthase subunit | X | X | X |
| *bdhA* D beta hydroxybutyrate dehydrogenase | ✓ | ✓ | ✓ |
| Phasin protein | ✓ | ✓ | ✓ |

**Phase Contrast- and Transmission Electron Microscopy**

For Phase Contrast Microscopy (PCM), a Nikon Eclipse Ci phase contrast microscope with digital camera (Nikon, UK) was used and samples prepared from agar plates. For Transmission Electron Microscopy (TEM), stationary phase cultures were centrifuged at 5,000 rpm for 5 minutes and resuspended in 0.1 M cacodylate buffer overnight then washed three times in the same buffer after which samples were re-suspended in 1 % aqueous osmium tetroxide and added to beam tubes. Cells were washed with water and then dehydrated with a graded ethanol series of 50, 70, 90, & 100 % ethanol, and then 100 % propylene oxide. Cells were infiltrated with epoxy resin overnight and then cells embedded by oven heating for 48 hours. Cell ultrathin sections (80 nm) were cut with a diamond knife using a Leica EM UC6 ultramicrotome, placed on copper grids, and then analysed using a Tecnai Bio-TWIN T12 Biotwin transmission electron microscope (TEM) (FEI company, Eindhoven, The Netherlands) and run at an accelerated voltage of 100 kV. Images were captured using a MegaView SIS camera, with representative images included in Figures.

**Phylogenetic analysis**

**Methods**: Data for 16S rRNA sequence of methanotrophs isolated in this study was obtained from the genome sequence assembled using Illumina and PacBio. For other species used for phylogenetic analysis, 16S rRNA data was obtained from Glöckner et al. (2017). FASTA sequences of 16S rRNA were uploaded to Molecular Evolutionary Genetics Analysis software (MEGA version 10) and aligned using Clustal Omega alignment tool (Kumar, 2018). Phylogenetic tree of the aligned sequences was obtained using Maximum Likelihood Tree as method of statistical analysis. Bootstrap method was used as Phylogeny test and statistical support for all trees was obtained from 1000 bootstrap replicates. Tamura-Nei was used as the substitution model and Nearest Neighbour Interchange was the Tree Interference option that was selected (Tamura and Nei, 1993).

**Whole Genome Alignment:** Whole genome alignment was carried out using a Muave Plugin (V1.1.1) on Geneious Prime. ProgressiveMauve algorithm was used to automatically calculate seed weight with Match Seed Weight value of 15. The minimum Locally Collinear Block (LCB) used for the alignment of *M. rosea* BRCS1 with *M. rosea* GW6 was 8814 whereas 22348 was used as the LCB for alignment of *M. parvus* OBBP with *M. parvus* BRCS2 (Darling et al., 2010). Average Nucleotide Identity was calculated using . Alignment options were minimum length of 700bp, minimum identity 70%, minimum alignment 50. For fragment options, window size was 1000bp while fragment size was 200bp (Rodrigueuz-R and Konstantinidis, 2015).

**Growth on different nitrogen sources**

Stationary phase pre-cultures of BRCS1 and BRCS2 were pelleted and re-suspended in nitrate free NMS. Isolates were inoculated into a 24 well plate in 1 mL of media in triplicate with the following nitrate sources at 0.05 % (w/v): Potassium nitrate, ammonium chloride, L-aspartate, L-lysine, L-asparagine, and L-glutamine. In addition, standard NMS (potassium nitrate 0.1 %) and nitrate free NMS were used as controls. Plates were incubated with methane at 30°C at 200 rpm in a gas tight box (EnzyScreen, NL). Methane was refreshed after 4 and 8 days and OD_600_ was recorded after 14 days. Nitrate free negative controls that showed final OD_600_ growth of around 0.01-0.06 were subtracted from the end results to give the final change in OD_600_. After isolation of methanotrophs, each strain was subjected to growth in different mineral salt media to investigate the preferred source of nitrogen. Standard NMS medium is prepared with 0.1 % potassium nitrate media although results in Figure S2 show that methanotrophs can use a variety of compounds as nitrogen source.


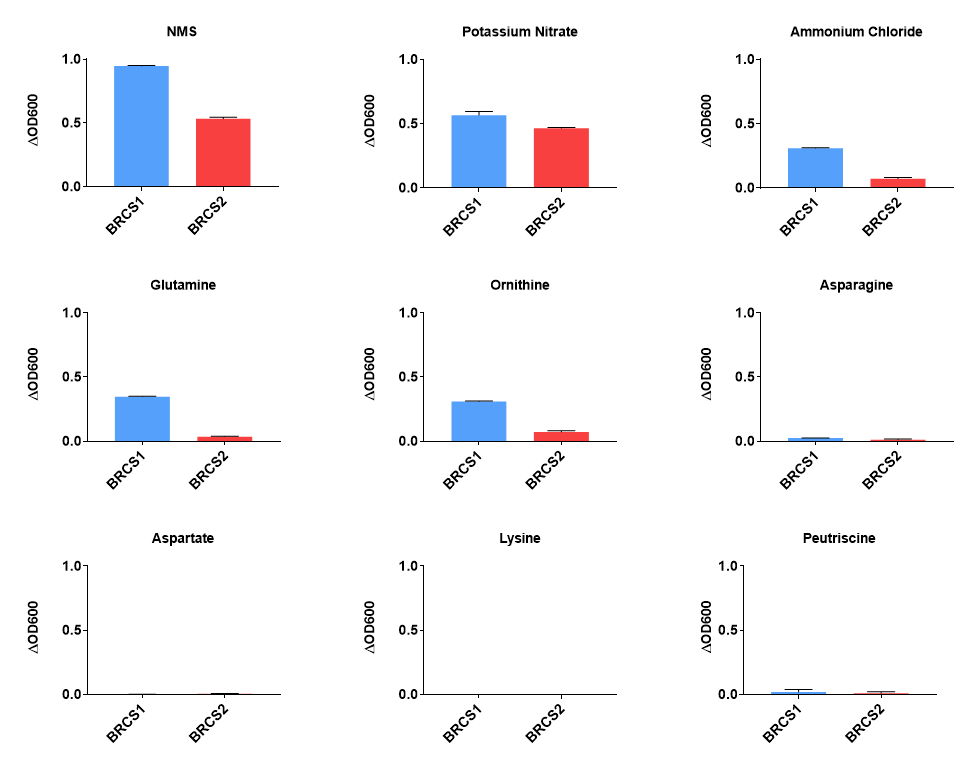


**Figure S2**. Isolates BRCS1 and BRCS2 were tested for growth on different nitrate sources. Both isolates were able to grow well on NMS (0.1% potassium nitrate) and NMS (0.05% potassium nitrate). They were not able to grow on asparagine, aspartate, lysine or putrescine

**Siloxane composition of AD gases**

Trace gases such as siloxanes, NH_3_ and H_2_S measured by Lucideon, an analytical company, are shown in Table S4.

**Table S4**. Trace gas composition of AD1 and AD2.

| Siloxanes [mg/m^3^] | AD1 | AD2 |
| --- | --- | --- |
| Hexamethylcyclotrisiloxane | n/a | < 1.0 |
| Octamethylcyclotetrasiloxane | n/a | 1.1 |
| Decamethylcyclopentasiloxane | 0.96 | < 1.0 |
| Hexamethyldisiloxane | n/a | < 1.0 |
| Octamethyltrisiloxane | n/a | < 1.0 |
| Decamethyltetrasiloxane | n/a | < 1.0 |
| Dodecamethylpentasiloxane | n/a | < 1.0 |
| Dodecamethylcyclohexasiloxane | 0.75 | < 1.0 |
| Trimethyl silanol (toluene equivalent) | n/a | < 1.0 |
| *N*,*N*-dimethyl acetamide | 2.4 | n/a |

n/a= not applicable (below detectable limit)

**Preliminary PHB accumulation assay**

One 250 mL serum bottle with 40 mL NMS medium and gassed with 29 mL CH_4_ and 71 mL air (additionally to existing headspace air) was inoculated with *Methylocystis parvus* OBBP and grown for 3 days shaking at 200 RPM and 30°C. The culture was used to inoculate twelve 250 mL serum bottles with 40 mL NMS to OD_600_ of 0.05 and gassed with 29 mL CH_4_ and 71 ml air every day during following growth. Headspace was flushed with sterile air before re-gassing to replenish headspace oxygen. After 4 days of growth, the cultures were spun down and resuspended in NMS without nitrate (apart from two control cultures which were resuspended in NMS with nitrate). Every day, 2 serum bottles were sacrificed for PHB analysis, the control culture was sacrificed at the end.

**References**

(1) Bankevich, A.; Nurk, S.; Antipov, D.; Gurevich, A. A.; Dvorkin, M.; Kulikov, A. S.; Lesin, V. M.; Nikolenko, S. I.; Pham, S.; Prjibelski, A. D.; et al. SPAdes: A New Genome Assembly Algorithm and Its Applications to Single-Cell Sequencing. *J. Comput. Biol.* **2012**, *19* (5), 455–477. https://doi.org/10.1089/cmb.2012.0021.

(2) Bolger, A. M.; Lohse, M.; Usadel, B. Trimmomatic: A Flexible Trimmer for Illumina Sequence Data. *Bioinformatics* **2014**, *30* (15), 2114–2120. https://doi.org/10.1093/bioinformatics/btu170.

(3) Chin, C. S.; Alexander, D. H.; Marks, P.; Klammer, A. A.; Drake, J.; Heiner, C.; Clum, A.; Copeland, A.; Huddleston, J.; Eichler, E. E.; et al. Nonhybrid, Finished Microbial Genome Assemblies from Long-Read SMRT Sequencing Data. *Nat. Methods* **2013**, *10* (6), 563–569. https://doi.org/10.1038/nmeth.2474.

(4) Darling, A. E; Mau, E., Perma, N. T. progressiveMauve: Multiple Genome Alignment with Gene Gain, Loss and Rearrangement. *Plos One* **2010.** https://doi.org/10.1371/journal.pone.0011147.

(5) Glöckner, F. O.; Yilmaz, P.; Quast, C.; Gerken, J.; Beccati, A.; Ciuprina, A.; Bruns, G.; Yarza, P.; Peplies, J.; Westram, R.; et al. 25 Years of Serving the Community with Ribosomal RNA Gene Reference Databases and Tools. *Journal of Biotechnology*. Elsevier B.V. November 10, 2017, pp 169–176. https://doi.org/10.1016/j.jbiotec.2017.06.1198.

(6) Kumar, S.; Stecher, G.; Li, M.; Knyaz, C.; Tamura, K. MEGA X: Molecular Evolutionary Genetics Analysis across Computing Platforms. *Mol. Biol. Evol.* **2018**, *35* (6), 1547–1549. https://doi.org/10.1093/molbev/msy096.

(7) Roberts, R. J; Vincze, T.; Posfai, J. and Macelis, D. REBASE—a Database for DNA Restriction and Modification: Enzymes, Genes and Genomes. *academic.oup.com* **2010**.

(8) Rodriguez-R, L.M. and Konstantinidis, K.T. The enveomics collection: a toolbox for specialized analyses of microbial genomes and metagenomes. **2016**. (No. e1900v1) PeerJ Preprints.

(9) Sambrook, J.; Fritsch, E. F.; Maniatis, T. *Molecular Cloning: A Laboratory Manual*; Cold Spring Harbor Laboratory, 1989.

(10) Seemann, T. Prokka: Rapid Prokaryotic Genome Annotation. *Bioinformatics* **2014**, *30* (14), 2068–2069. https://doi.org/10.1093/bioinformatics/btu153.

(11) Simon, R.; Priefer, U.; Pühler, A. A Broad Host Range Mobilization System for in Vivo Genetic Engineering: Transposon Mutagenesis in Gram Negative Bacteria. *Bio/Technology* **1983**, *1* (9), 784–791. https://doi.org/10.1038/nbt1183-784.

(12) Tamura, K.; Nei, M. Estimation of the Number of Nucleotide Substitutions in the Control Region of Mitochondrial DNA in Humans and Chimpanzees. *Mol. Biol. Evol.* **1993**. https://doi.org/10.1093/oxfordjournals.molbev.a040023.
